# A comparative study of dentinal tubule penetration and the retreatability of EndoSequence BC Sealer HiFlow, iRoot SP, and AH Plus with different obturation techniques

**DOI:** 10.1007/s00784-020-03747-x

**Published:** 2021-02-26

**Authors:** Ruiqi Yang, Jun Tian, Xiangya Huang, Shuxiang Lei, Yanling Cai, Zhezhen Xu, Xi Wei

**Affiliations:** 1 Department of Operative Dentistry and Endodontics, Guanghua School of Stomatology, Hospital of Stomatology Sun Yat-sen University, 56 Ling Yuan Xi Road, Guangzhou, 510055 Guangdong China; 2 Guangdong Province Key Laboratory of Stomatology, Sun Yat-sen University, Guangzhou, Guangdong China

**Keywords:** BC Sealer HiFlow, Dentinal tubule penetration, iRoot SP, Obturation technique, Retreatability

## Abstract

**Objectives:**

This study aimed to evaluate dentinal tubule penetration and the retreatability of EndoSequence BC Sealer HiFlow (HiFlow), iRoot SP, and AH Plus when using the single-cone (SC) or continuous wave condensation (CWC) technique.

**Materials and methods:**

Sixty-five single-rooted teeth were instrumented and randomly divided into 5 groups: group 1, AH Plus/CWC; group 2, iRoot SP/CWC; group 3, iRoot SP/SC; group 4, HiFlow/CWC; and group 5, HiFlow/SC. The ability to re-establish patency during endodontic retreatment was recorded, as was the time taken to reach the working length. Dentinal tubule penetration and remaining debris after retreatment were evaluated by confocal microscopy and scanning electron microscopy. Data were analyzed by Kruskal-Wallis test and Dunn’s multiple comparisons test (*α* = 0.05).

**Results:**

The HiFlow/CWC and iRoot SP/CWC groups required more time to reach the working length than groups that underwent the SC technique regardless of the sealer used (*P* < .05). The HiFlow/CWC group showed a significantly higher percentage of sealer penetration area than that of the iRoot SP/SC at 4 mm from the apex (*P* < .05) and penetrated deeper into dentinal tubules than iRoot SP/SC at both 8-mm and 12-mm levels (*P* < .05). Moreover, the HiFlow/CWC and HiFlow/SC groups demonstrated less remaining sealer along the canal wall than AH Plus/CWC group at 4-mm level (*P* < .05).

**Conclusions:**

HiFlow/CWC technique showed better performance in dentinal tubule penetration than that of iRoot SP/SC. Both HiFlow and iRoot SP combined with CWC technique groups required more retreatment time than the other groups. Furthermore, using HiFlow with either the CWC or SC technique left less remaining sealer at 4-mm level than using AH Plus with the CWC technique during retreatment.

**Clinical relevance:**

With favorable performance in dentinal tubule penetration and retreatability in endodontic retreatment, the combined use of EndoSequence BC Sealer HiFlow with the recommended continuous wave condensation technique may be a worthwhile choice in root canal treatment.

## Introduction

The calcium silicate–based sealer iRoot SP (Innovative BioCreamix Inc., Vancouver, Canada), also named Endosequence BC Sealer (Brassiere, Savannah, Georgia, USA), has attracted considerable attentions due to its good biocompatibility, bioactivity, sealing ability, osteoconductive effects [1–3], and ability to chemically bond to root canal dentin [4]. It is a premixed, injectable material composed of zirconium oxide, calcium silicates, calcium phosphate, calcium hydroxide, filler, and thickening agents [5]. iRoot SP has favorable flowability, small particle size, and no setting shrinkage, as well as volume expansion to some extent [6, 7]; thus, the manufacturer has recommended using the single-cone (SC) technique for iRoot SP.

Sealer penetration into dentinal tubules by mechanical locking and chemical bonding [8] forms a physical barrier, improves the retention of the root canal filling, and entombs residual bacteria [8–10]. On the other hand, a desirable property of an ideal root filling material or sealer outlined by Grossman [11] was the ability to be easily removed from the root canal if necessary. Retreatment consists of the removal of existing filling material to allow disinfection of the root canal system to promote periapical healing [12]. Several parameters have been used to assess the retreatability of sealers, including the ability to regain the working length (WL) and patency, the time to reach the WL, and the amount of remaining root canal filling material or debris [5, 13, 14]. Studies on the dentinal tubule penetration and retreatability of iRoot SP have led to inconsistent results since different obturation techniques have been used [14–17]. It was reported that iRoot SP with the continuous wave condensation (CWC) technique was more effective at filling artificial lateral canals than the SC technique [18]. However, recent studies demonstrated that heat affected the physical properties and chemical composition of sealers [19–22]. A previous study demonstrated that AH Plus showed acceptable changes in physical properties at a high temperature, while iRoot SP showed significant reductions in setting time and flow [23]. The changes in sealers induced by high temperature may affect the quality of obturation during warm vertical compaction.

To overcome the quality constraint resulting from high temperature, a novel premixed bioceramic sealer called EndoSequence BC Sealer HiFlow (Brasseler, Savannah, Georgia, USA) has been introduced recently. According to the manufacturer’s claims, BC Sealer HiFlow exhibits a lower viscosity when heated and is more radiopaque than BC Sealer, making it optimized for warm obturation techniques. Recent studies demonstrated that HiFlow had similar cytocompatibility and bioactivity to that of BC Sealer [24], while it showed better performance on flow than BC Sealer when the warm vertical compaction technique was used [25]. However, there are no published data on the dentinal tubule penetration of BC Sealer HiFlow using different obturation techniques. Likewise, the retreatability of the sealer remains unknown.

Therefore, the aim of this study was to evaluate and compare the dentinal tubule penetration and retreatability of BC Sealer HiFlow, iRoot SP, and AH Plus with the CWC and SC techniques.

## Materials and methods

### Sample size calculation

Based on the data of two previous studies [5, 26], the sample size in the present study was calculated by the PASS 11 software (Power Analysis & Sample Size, NCSS, USA). In the ANOVA study, sample sizes of 10, 10, 10, 10, and 10 were obtained from the 5 groups. The total sample of 50 subjects achieves 81% power to detect differences with a 0.05000 significance level.

### Specimen preparation

Sixty-five single-rooted human premolars extracted for orthodontic reasons were collected under a protocol approved by the Research Ethics Committee of Sun Yat-sen University. Preoperative radiographs were taken in the buccolingual and mesiodistal directions to confirm the presence of a single canal and to select teeth with a long to short diameter ratio of ≤ 2.5 at 5 mm from the apex. Roots with curvatures higher than twenty degrees [5], immature apices, fractures, calcification, resorption, previous endodontic treatment, or initial apical sizes larger than 20 were rejected. All teeth were decoronated using a water-cooled diamond bur. The WL was determined by subtracting 0.5 mm from the length of a size 10 K-file (Dentsply Maillefer) until the tip of the instrument became visible at the apical foramen. The canals were instrumented using the ProTaper NEXT (PTN) rotary system (Dentsply Maillefer) to a size 30 07 taper (X3) following the manufacturer’s instructions. After each instrument was used, the canals were irrigated with 2 mL 3% sodium hypochlorite (NaOCl) and saline solution with a 30-G needle. Then, passive ultrasonic irrigation with 3% sodium hypochlorite was performed as described by van der Sluis et al. [27]. After irrigation with saline solution, 10 mL 17% EDTA solution was applied for 60 s to remove the smear layer. Finally, the canals were flushed with saline solution and dried with paper points (size 25, Dentsply Maillefer).

The specimens were randomly divided into five experimental groups based on sealers and obturation techniques (each group, n = 13) as follows:

1. AH Plus with the CWC technique (AH Plus/CWC)
2. iRoot SP with the CWC technique (iRoot SP/CWC)
3. iRoot SP with the SC technique (iRoot SP/SC)
4. BC Sealer HiFlow with the CWC technique (HiFlow/CWC)
5. BC Sealer HiFlow with the SC technique (HiFlow/SC)

Sealers were mixed with rhodamine B (Sigma-Aldrich, St. Louis, MO) for fluorescence at a 100:1 ratio by weight [15]. All of the sealers were prepared according to the manufacturer’s instructions. For groups using the CWC technique, a master cone (size 30 07 taper) (Dentsply Maillefer) coated with sealer was inserted into the root canal with tug-back at the WL. The heated plugger at 200 °C with an appropriate tip was used to leave 4 mm of gutta-percha in the apical third by the obturation unit (SybronEndo, Kerr Endodontics, Orange, CA, USA). The remaining middle and coronal thirds were backfilled with thermoplasticized gutta-percha. For groups using the SC technique, sealer mixed with rhodamine B was injected into the inner canal. The master cone (size 30 07 taper) was coated with mixed sealer and inserted into the root canal until the WL was reached. The access cavities were temporarily sealed with Caviton (GC, Tokyo, Japan) with a minimum thickness of 3.5 mm [28]. Radiographs were taken to confirm the quality of obturation. The specimens were stored in a humidified chamber (100% humidity and 37 °C) for 7 days to allow the sealers to set completely.

The root fillings were removed with ProTaper universal retreatment files D1, D2, and D3 (Dentsply Maillefer), and the root canals were prepared with a PTN rotary system according to the manufacturer’s instructions without using solvent. The measurement of the time taken to reach the WL began with the use of D1 and ended when a size 40 06 taper (X4) instrument reached the WL [29]. The canals were irrigated with 3% NaOCl between instruments. All endodontic procedures were performed by the same operator. In each group, 3 teeth were randomly selected for scanning electron microscopy (SEM) examination and the other 10 teeth were analyzed by confocal laser scanning microscopy (CLSM).

### Confocal laser scanning microscopy

Teeth were embedded in wax and sectioned horizontally 4 mm, 8 mm, and 12 mm from the apex with a thickness of 100 μm per section at a slow speed (Accutom-50, Struers, UK) under water-cooling conditions. Specimens (each group, *n* = 10) were scanned under a CLSM (LSM 800; Zeiss, Germany). Photographs taken 10 μm below the surface with Zen 2012 (Zeiss) software were analyzed using ImageJ software. The ImageJ software was used to measure the depth and areas of sealer, as well as the total canal space. This program can calculate area and pixel value statistics of user-defined selections and intensity thresholded objects. The CLSM evaluation included the following three indicators [15]: the sealer penetration depth, which was measured from the canal wall to the point of maximum sealer penetration (mm); the sealer penetration area, which was calculated as the sealer penetration area into the dentinal tubules divided by the horizontal root section area × 100 (%); and the amount of sealer remaining in the canal space, which was calculated as the amount of sealer in the canal space divided by the total canal space × 100 (%).

### Scanning electron microscopy

Canal cleanliness after retreatment was examined by SEM (Quanta 200; FEI, Czech). Specimens were embedded in wax and sectioned longitudinally by a slow-speed saw under water-cooling conditions. After coating with gold by ion sputtering (Eiko IB-5; Eiko Engineering Co Ltd, Hitachinaka, Japan), specimens (each group, *n* = 3) were examined and photographed at an acceleration voltage of 20 kV at various magnifications. The debris particles, probably constituted by sealers, had a diameter of 5–6 μm while gutta-percha debris was frequently represented by small fragments of 20–30 μm [30].

### Statistical analysis

IBM SPSS v.20 (NY, USA) was used for statistical analysis. Because of the absence of a normal distribution, statistical analysis was performed by using the nonparametric Kruskal-Wallis test and Dunn’s multiple comparisons test for retreatment time, sealer penetration depth, sealer penetration area, and the remaining sealer along canal wall. The significance level for these tests was set at *α* = 0.05.

## Results

### Patency re-establishment and the time taken to reach the working length

**Fig. 1:**
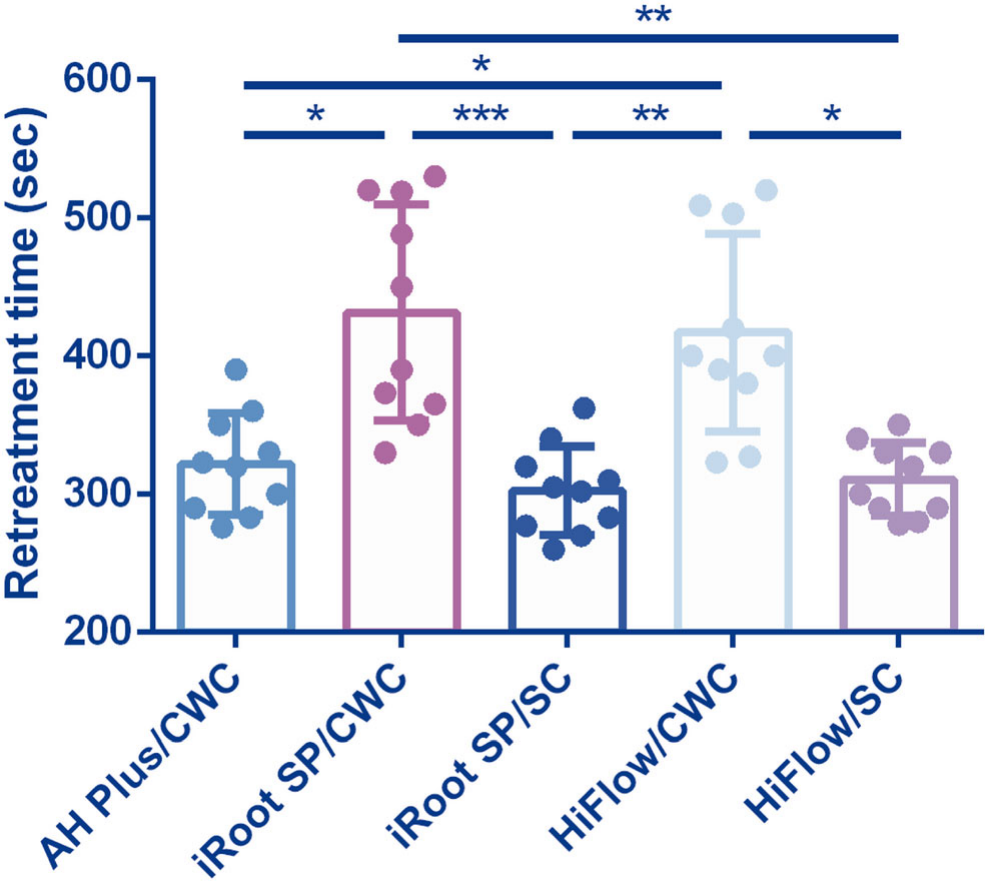
The time taken to re-establish patency and to reach the WL (seconds) in five groups (*P < .05, **P < .01, and ***P < .001)

Canal patency was achieved in every specimen. The time taken to reach the WL of the five groups is shown in Fig. 1. Both the iRoot SP/CWC and HiFlow/CWC groups required more time to reach the WL than the iRoot SP/SC, HiFlow/SC, and AH Plus/CWC groups. No significant differences were detected between the iRoot SP/SC and HiFlow/SC groups regarding the time taken to reach the WL.

### Sealer penetration into dentinal tubules

**Fig. 2:**
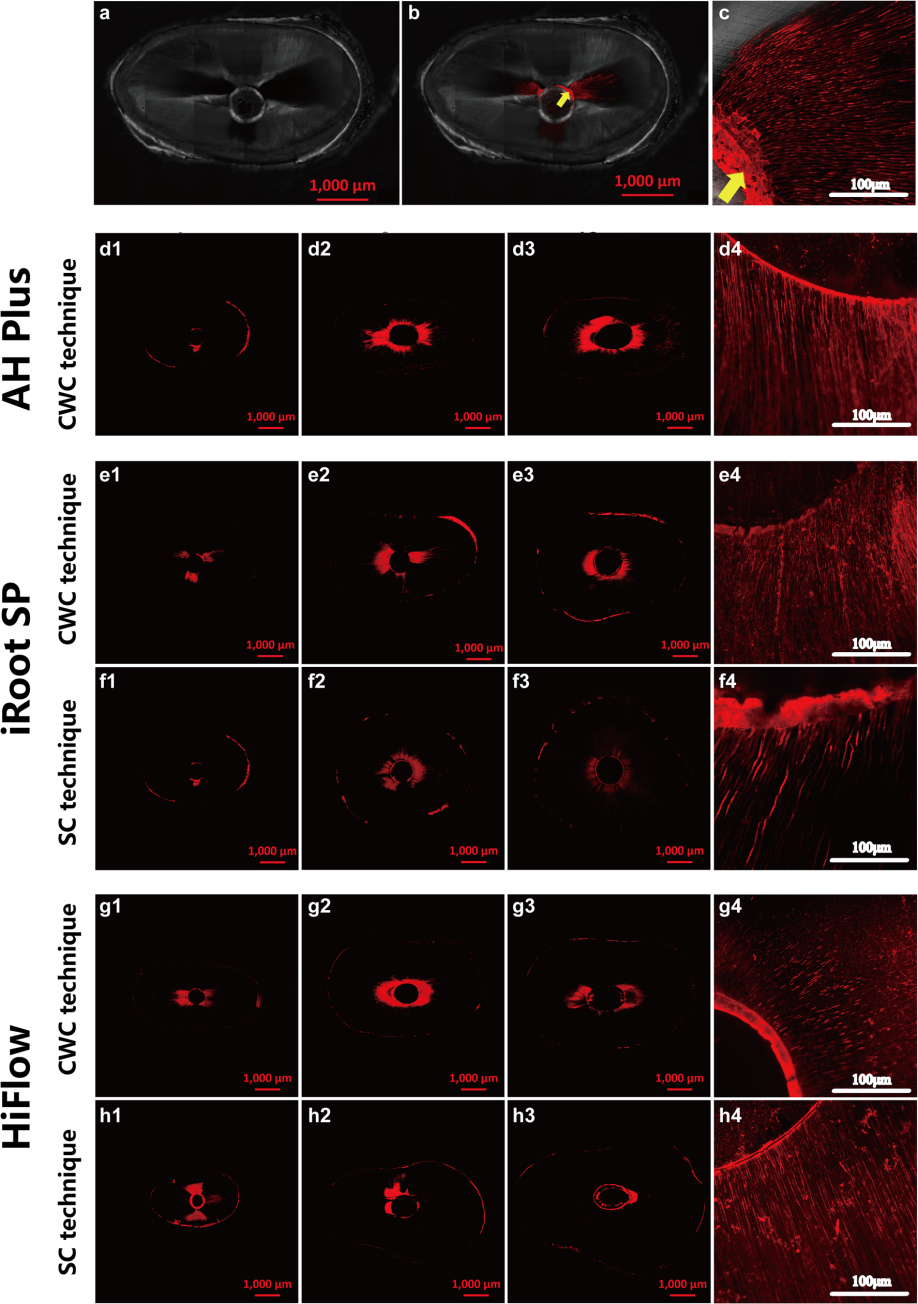
Representative confocal laser scanning microscopy (CLSM) images of resected root surfaces at the 4-mm, 8-mm, and 12-mm levels from the apex after retreatment in five groups. (a) A scanned image of a resected root surface. (b) A confocal microscopy image overlapping with (a) shows sealers in the canal (yellow arrow) and sealer penetration into dentinal tubules, with the area of sealer mixed with rhodamine B shown in red. (c) A higher magnification image shows sealers remaining in the canal (yellow arrow). (d1–d4) AH Plus/CWC group. (e1–e4) iRoot SP/CWC group. (f1–f4) iRoot SP/SC group. (g1–g4) HiFlow/CWC group. (h1-h4) HiFlow/SC group

**Fig. 3:**
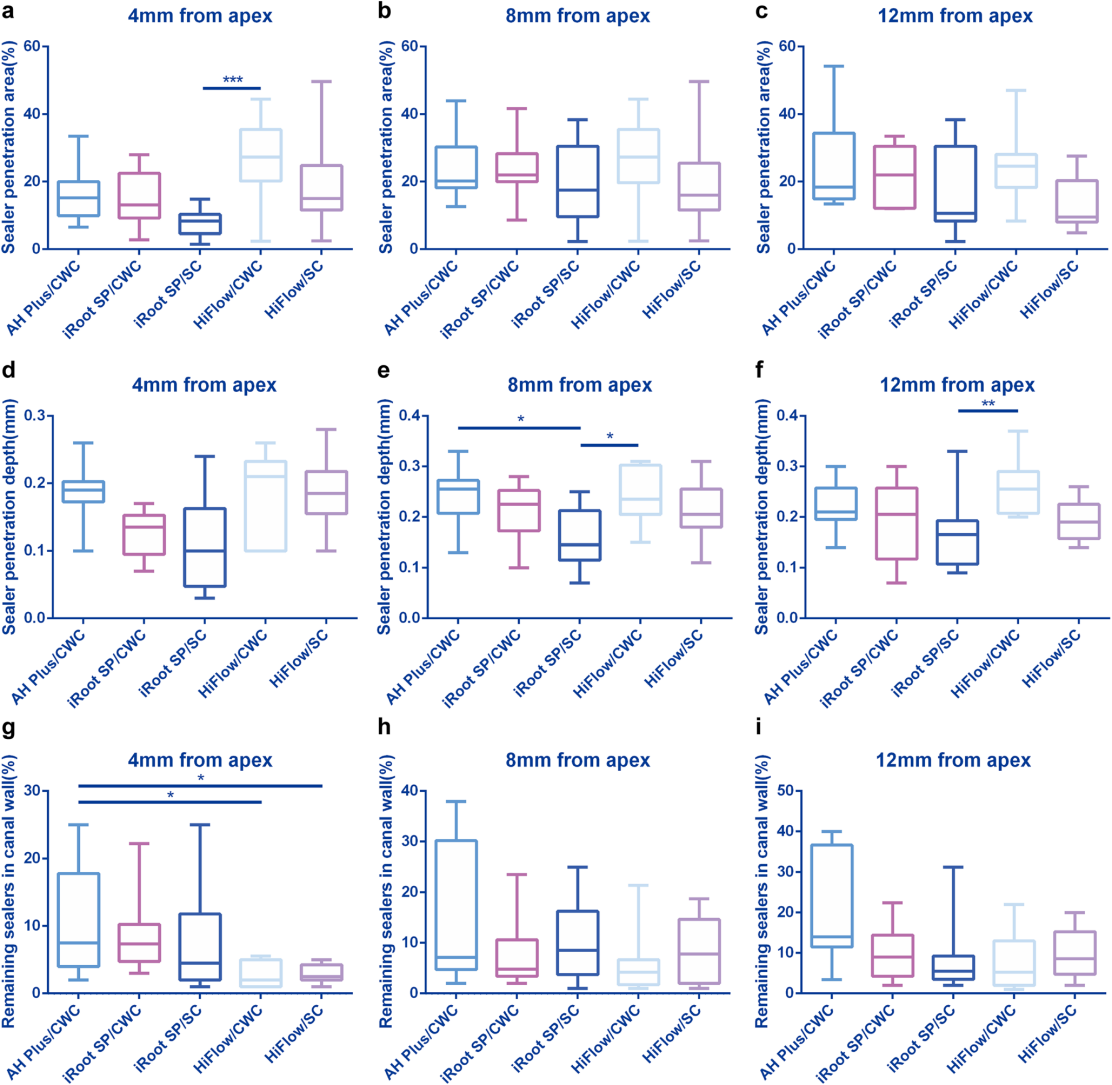
Dentinal tubule penetration and remaining sealers in the canal wall in the five groups. (a–c) Box plots of the sealer penetration area (%) at the 4 mm, 8 mm, and 12 mm from the apex. (d–f) Box plots of the sealer penetration depth at the 4 mm, 8 mm, and 12 mm from the apex. (g–i) Box plots of the remaining sealers in canal wall (%) at the 4 mm, 8 mm, and 12 mm from the apex (*P < .05, **P < .01, and ***P < .001)

Representative images under CLSM are shown in Fig. 2. The sealer penetration areas (%) in the five groups are shown in Figure 3a–c. A statistically significant difference was observed only at 4-mm level between the HiFlow/CWC and iRoot SP/SC groups (*P* < .05).

The sealer penetration depths at the 4-mm, 8-mm, and 12-mm levels are shown in Fig. 3d–f. The HiFlow/CWC group exhibited deeper penetration into dentinal tubules than that of the iRoot SP/SC group both at the 8-mm and 12-mm levels (*P* < .05). The AH Plus/CWC group also showed significantly deeper penetration than that of the iRoot SP/SC at the 8-mm level (*P* < .05).

### The amount of remaining sealer along canal wall (%)

The HiFlow/CWC and HiFlow/SC groups showed less remaining sealer in root canal walls than that of the AH Plus/CWC group at the 4-mm level (*P* < .05) (Fig. 3g). There were no significant differences among groups at the 8-mm and 12-mm levels (Fig. 3h and i).

**Fig. 4:**
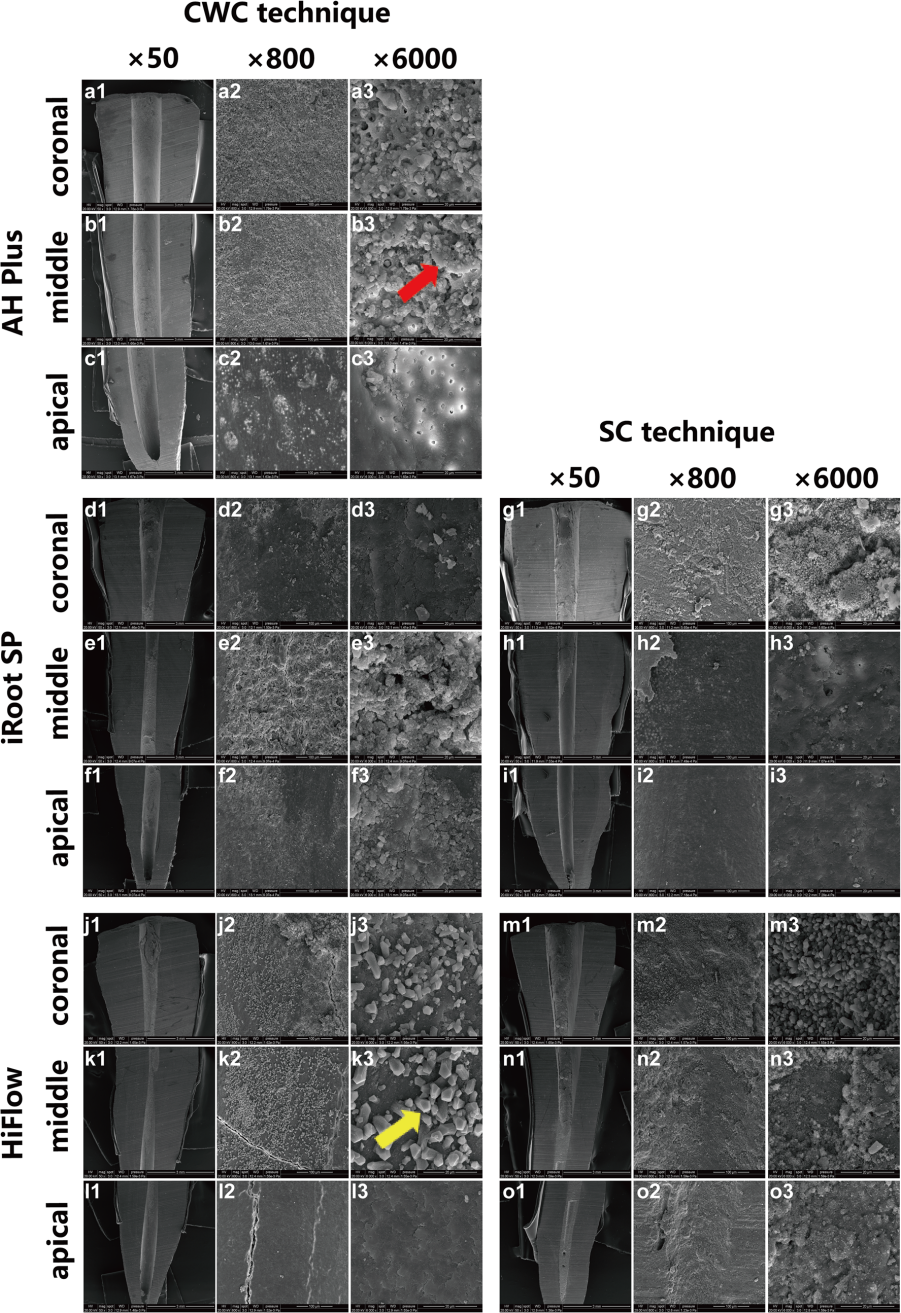
Scanning electron microscopy (SEM) images of retreated root canals in the coronal, middle, and apical thirds with 3 different magnifications ([a1–o1] × 50, [a2–o2] × 800, [a3–o3] × 6000) in five groups. The red arrow shows the gutta-percha debris with a diameter of 20–30 μm (b3). The yellow arrow shows sealers debris with a diameter of 5–6 μm (k3)

Representative SEM images revealed that none of the techniques completely removed the sealer. Debris remained in the canal walls at 4-mm, 8-mm, and 12-mm levels in all groups (Fig. 4). High magnification images showed gutta-percha debris (Fig. 4b3) and sealer debris (Fig. 4k3).

## Discussion

Three-dimensional obturation of the root canal system is of vital importance for successful endodontic therapy [31]. The combination of gutta-percha and root canal sealer is suggested to provide an adequate seal for root canal obturation. Microleakage, which adversely affects the success of root canal therapy [32], could be influenced by root canal sealing [33]. Calcium silicate–based sealers possess high hydraulic conductance which tends to obstruct dentinal tubules [34]. Therefore, an assessment of the dentinal tubule penetration of sealers with different obturation methods could reflect their potential sealing effect in filled root canals. In this study, the novel calcium silicate–based sealer EndoSequence BC Sealer HiFlow was evaluated for dentinal tubule penetration. To our knowledge, this is the first in vitro study on the dentinal tubule penetration and retreatability of the novel EndoSequence BC Sealer HiFlow.

Sealer penetration is related to several factors, such as the flow of the sealer [17, 35], the number and diameter of tubules, and the obturation technique required [36]. The influential factors for the flow of the sealer include temperature, setting time, and particle size [37]. In the present study, the percentage of sealer penetration area in the HiFlow/CWC group was significantly higher than that in the iRoot SP/SC group at the 4-mm level. It was reported that the maximal temperature during warm vertical compaction in the root canal was 118 °C at the 8-mm level and 52 °C at the 4-mm level from the apex [38]. Another study showed that the maximal intracanal temperature increase was 19.2 °C (under a 37 °C environment) at the 6-mm level using CWC, with no significant difference between the 3-mm and 6-mm levels [39]. More recently, System B at 200 °C exhibited the highest temperature at the 12-mm level, followed by the 2-mm and 8-mm levels, with a range of 40~60 °C [40]. Taken together, the intracanal temperature changes appeared to be limited during CWC. Another discrepant factor between the CWC and SC techniques that may affect dentinal penetration was compaction. However, one study demonstrated that the dentinal penetration depth of calcium silicate–based sealers at the 4-mm level from the apex was not affected by the extra pressure created by warm vertical compaction [33]. Therefore, the difference in dentin penetration between the HiFlow/CWC and iRoot SP/SC groups at the 4-mm level might be attributed to the flow of the sealers rather than the obturation technique used. According to a recent study [25], HiFlow had higher flow than iRoot SP at both room temperature and high temperature, while the setting times of the two sealers were similar at 37 °C and 100 °C. Notably, both HiFlow and iRoot SP are premixed calcium silicate–based sealers, which have major inorganic components including C_3_S, C_2_S, and calcium phosphates. This indicates that the two sealers have similar particle sizes. The present study appears to indicate that BC Sealer HiFlow with the matched CWC technique may achieve better sealing ability than iRoot SP with the recommended SC technique in the apical third. As an adequate seal of the apical third is essential for the placement of a post and core [41], BC Sealer HiFlow in combination with the CWC technique may represent a noteworthy alternative to iRoot SP with the SC technique when an intracanal post is indicated following obturation. Further studies are required to confirm this speculation. In the middle and coronal thirds with an increasing number and diameter of dentinal tubules, a significant difference was found in the penetration depth rather than in the penetration area between the HiFlow/CWC and iRoot SP/SC groups. In addition to the difference in sealer flow, the compaction force during the CWC technique may be the other contributing factor, as the AH Plus/CWC group tended to demonstrate deeper penetration than that of the iRoot/SC group at the 8-mm level as well. Taken together, HiFlow with the CWC technique had better performance in sealer penetration along the root canal than iRoot SP with the SC technique, which may result in better root canal sealing and improve the obturation outcome.

Persistent infections are considered the most common cause of root canal failures [42]. Re-establishing patency and WL in retreatment cases is fundamental for successful retreatment [43]. In the present study, the retreatability of calcium silicate–based sealers was investigated and patency was achieved in every specimen. Previous studies have compared the retreatment time of calcium silicate–based sealers with that of epoxy resin-based sealers with different obturation techniques but failed to draw a concrete conclusion. To our knowledge, there have been no comparative studies on the retreatment time between the SC and CWC techniques using the same calcium silicate–based sealer. In the present study, the HiFlow/CWC and iRoot SP/CWC groups required a significantly longer time to reach the WL than the groups using the SC technique. Kim et al. commented that calcium silicate–based sealers used in the CWC technique would have larger volumetric ratio of gutta-percha than the SC technique and thus might have better retreatability [44]. However, we found that the removal of gutta-percha from canals filled with the CWC technique was more time-consuming than the removal of a single gutta-percha point in the SC group. In addition, calcium silicate–based sealers with the CWC technique required much more time than the AH Plus/CWC group, which was consistent with some previous studies [5, 45]. This might be because the dentine bond strength of bioceramic sealers is higher than that of resin-based sealers [4, 46]. The main components of bioceramic sealers—calcium silicate and calcium phosphate—could form a chemical bond with the root canal, reducing the occurrence of microleakage [14]. However, the dentine bond strength also increases the difficulty in removing the sealers during retreatment. In addition, the bioceramic sealers become very hard to penetrate after they have been allowed to set completely [5]. Eymirli et al. [47] demonstrated that working length could not be achieved during retreatment in root canals obturated with sealers only.

Previous studies have reported that the majority of the remaining filling material on the canal walls was sealer based [48]. Adequate removal of the sealer is essential during retreatment procedures to establish healthy periapical tissues [49]. Two studies previously found that the use of solvents, such as chloroform and Endosolv, in the early stages of instrumentation could reduce the time to reach the working length but does not facilitate root canal cleanliness [50, 51]. Ethylene diamine tetracetic acid (EDTA) is a calcium chelator and is usually employed for smear layer removal in endodontics [52]. A recent preliminary study proved that the 17% EDTA used in combination with mechanical cleaning was more effective in the removal of bioceramic-coated gutta-percha than conventional gutta-percha cone and sealer [53]. Moreover, 10% formic acid in conjunction with mechanical instrumentation could effectively remove conventional gutta-percha and sealer, as well as the bioceramic-coated gutta-percha [53]. So, the use of 17% EDTA or 10% formic acid in combination with proper mechanical instrumentation could help promote the removal of gutta-percha and sealer. The instruments used in root canal retreatment also play a key role in the outcome of non-surgical endodontic retreatment. According to a systematic review [54], reciprocating and rotary systems exhibited similar potential in removing root filling materials, while solvents hindered root canal cleaning. The PTN and ProTaper universal retreatment systems showed favorable effectiveness in removing filling material [55–57]. Therefore, in the present study, we used ProTaper universal retreatment files and a PTN rotary system without solvent during retreatment. The master apical file (MAF) for retreatment was one size larger (X4, size 40 06 taper) than MAF (X3, size 30 07 taper) for initial treatment based on a previous study [29] to remove more filling materials. Our findings, in accordance with previous studies, showed that conventional retreatment techniques failed to fully remove calcium silicate–based or epoxy resin-based sealers [5, 14]. Furthermore, a study showed that more filling material, including sealers and gutta-percha, remained in the apical third after retreatment [58]. Cleaning of the apical third has become a challenge in root canal retreatment. When we evaluated the remaining sealer among different groups in this study, we found that less sealer remained when using BC Sealer HiFlow than when using AH Plus at the 4-mm level regardless of the obturation techniques. This may be related to the low film thickness of HiFlow at both room and high temperatures [25], which is regarded as an important physical property for sealers. Previous studies indicated that film thickness and the flow of the sealers were related to the ability to fill root canals successfully [59, 60]. Our study further suggested that sealers characterized by lower film thickness and a better flow ability might present improved convenience regarding removal for retreatment cases.

Three-dimensional obturation of irregular root canals with complex anatomy has been a challenge in endodontics, as well as root canal retreatment if necessary. Based on the present study, the combination use of BC Sealer HiFlow with the CWC technique seemed to exhibit better dentinal tubule penetration and retreatability, which may be advantageous for irregular root canals to achieve better apical sealing, as well as better root canal cleaning in case of retreatment. Besides, BC Sealer HiFlow was proved to have favorable biological properties and promote expressions of oste/cementogenic genes by human periodontal ligament stem cells [24]. These characteristics may make the combination use of BC Sealer HiFlow with CWC technique more beneficial for irregular root canals with apical lesions. Further studies are needed to confirm this speculation.

To evaluate dentinal tubule penetration or remaining debris in root canal, various methods have been applied, including micro computerized tomography (micro CT), stereomicroscopy, SEM, and CLSM. Micro CT is a non-destructive method which provides 3D images with high accuracy and spatial resolution [61]. It is usually used to detect dentine defects such as cracks or to measure the volumes of filling material in root canal [62–64], rather than the sealers penetration in the dentinal tubules or remaining sealers along canal wall. In a study using stereomicroscopy method for evaluation, sealer could only be classified or scored based on the presence of the material [14]. Various studies have applied CLSM and SEM for evaluations of sealer penetration into dentinal tubules or remaining sealers [5, 15, 33, 47, 65]. In the present study, CLSM was applied to effectively evaluate the depth and areas of sealer penetration into dentinal tubules at different levels, as well as the remaining sealer after retreatment. SEM was applied for direct morphology of residual debris in the root canal. The combination of CLSM and SEM provided both quantitative and morphological evaluations of representative samples. However, in order to reduce the influence of fluorescence intensity on quantitative evaluation, samples need to be evaluated with CLSM in short time. A limitation for SEM is the difficulty of sample preparation, including longitudinal section of samples to expose the entire root canal.

The manufacturers claimed that BC cones, which are impregnated and coated with bioceramic particles, would allow for chemical bonding with BC Sealer. Recently, a new BC Points 150 series was recommended for combined use with BC Sealer HiFlow. In this study, we used gutta-perchas matched for nickel titanium rotary instruments to mimic most clinical situations, similar to many previous studies of BC Sealer or iRoot SP [4, 5, 14]. It would be interesting to observe the potential advantages when BC Points 150 series are used with BC Sealer HiFlow. Further studies are required to evaluate the apical sealing ability and clinical performance of the novel sealer. A limitation of an in vitro study lies in the fact that although a balanced distribution was attempted to be achieved in terms of the dimensions of the experimental teeth, standardization is difficult to be provided in terms of the number of dentinal tubules as well as their areas. Another limitation is that the canal anatomy in clinical cases would be much more complex than single root teeth used in this study. Nevertheless, our study may lay foundation for further study using teeth with more complex anatomy.

## Conclusions

Within the limitations of this study, BC Sealer HiFlow with the CWC technique showed better performance in dentinal tubule penetration than iRoot SP with the SC technique. Both the BC Sealer HiFlow and iRoot SP combined with the CWC technique required more time to re-establish patency and to reach the WL than the other evaluated combinations required. BC Sealer HiFlow, with the CWC or SC technique, had less remaining sealer during retreatment in the apical third than AH Plus with the CWC technique during retreatment.

Based on the relatively excellent dentinal tubule penetration and retreatability, the combined use of BC Sealer HiFlow with the recommended CWC technique may be a worthwhile choice in root canal treatment.
